# Effects of *Caragana korshinskii* tannin on fermentation, methane emission, community of methanogens, and metabolome of rumen in sheep

**DOI:** 10.3389/fmicb.2024.1334045

**Published:** 2024-02-15

**Authors:** Xiaoyu Niu, Yuanyaun Xing, Jingyao Wang, Lili Bai, Yongfang Xie, Shouqian Zhu, Mei Sun, Jing Yang, Dabiao Li, Yuanyuan Liu

**Affiliations:** ^1^Inner Mongolia Key Laboratory of Animal Nutrition and Feed Science, College of Animal Science, Inner Mongolia Agricultural University, Hohhot, China; ^2^College of Science, Inner Mongolia Agricultural University, Hohhot, China

**Keywords:** condensed tannin, sheep, nutrient digestibility, volatile fatty acids, methane emission

## Abstract

The purpose of this research was to investigate the impact of dietary supplementation of *Caragana korshinskii* tannin (CKT) on rumen fermentation, methane emission, *methanogen* community and metabolome in rumen of sheep. A total of 15 crossbred sheep of the Dumont breed with similar body conditions, were divided into three groups (*n* = 5), which were fed with CKT addition at 0, 2 and 4%/kg DM. The study spanned a total of 74 days, with a 14-day period dedicated to adaptation and a subsequent 60-day period for conducting treatments. The results indicated that the levels of ammonia nitrogen (NH_3_-N) and acetate were reduced (*p* < 0.05) in rumen sheep fed with 2 and 4% CKT; The crude protein (CP) digestibility of sheep in 2 and 4% CKT groups was decreased(*p* < 0.05); while the neutral detergent fiber (NDF) digestibility was increased (*p* < 0.05) in 4% CKT group. Furthermore, the supplementation of CKT resulted in a decrease (*p* < 0.05) in daily CH_4_ emissions from sheep by reducing the richness and diversity of ruminal *methanogens* community, meanwhile decreasing (*p* < 0.05) concentrations of tyramine that contribute to methane synthesis and increasing (*p* < 0.05) concentrations of N-methy-L-glutamic acid that do not contribute to CH_4_ synthesis. However, CH_4_ production of DMI, OMI, NDFI and metabolic weight did not differ significantly across the various treatments. To sum up, the addition of 4% CKT appeared to be a viable approach for reducing CH_4_ emissions from sheep without no negative effects. These findings suggest that CKT hold promise in mitigating methane emissions of ruminant. Further investigation is required to evaluate it effectiveness in practical feeding strategies for livestock.

## 1 Introduction

The problems of global warming and climate change have gained worldwide attention. According to Moss et al. (2000), the contribution of livestock to global anthropogenic methane (CH_4_) emissions is estimated as 14–18%. Moreover, the generation of CH_4_ through the process of carbohydrate fermentation in the rumen is an energy utilization inefficiency (Millen et al., 2016). Hence, it is highly important to decrease CH_4_ emissions from ruminants, as it not only helps in mitigating climate change but also improves the efficient utilization of feed in livestock production (Chen et al., 2015).

For years, plant secondary metabolites have been regarded as harmful to animals and were referred to as anti-nutritional factors (Sarwar et al., 2012). Nevertheless, over the past few decades, there has been an increasing interest with these metabolites in the field of animal nutrition. This interest stems from their potential to enhance rumen function and reduce CH_4_ emissions by engaging various mechanisms that involve the rumen microbiome (María et al., 2020). Among them, condensed tannins (CT) stand out as highly promising. Researches on CH_4_ inhibition methods associated with diets rich in tannins or tannin extracts have been conducted with sheep (Adejoro et al., 2019) and dairy cows (María et al., 2020). According to prior research, the characteristics of ruminant animals were influenced by the microorganisms in their rumen, and the functions of these microorganisms could be observed through the metabolites (Hua et al., 2017). Nevertheless, the majority of research has primarily concentrated on the alteration in rumen fermentation (Ogunade et al., 2019; Avila et al., 2020) or the digestion of nutrients (Wang et al., 2021). The rumen microbiome assumes a crucial function in all ruminant species, participating in their utilization of nutrients, detoxification, and emission of CH_4_. Rumen *methanogens* use the H_2_ resulting from the fermentation of carbohydrates to reduce CO_2_ to CH_4_ in a series of biochemical reactions (Kuvera et al., 2019). And few reports studied the effects of CKT on CH_4_ emission in sheep. Which may be one of the main reasons why CKT inhibit CH_4_ emissions, and further study is needed.

*Caragana korshinskii* (CK) is extensively cultivated in arid and semi-arid farming-pasture areas of Northwestern China. It is commonly employed as a shrub species for afforestation and to combat desertification due to its ecological significance (Long et al., 2020). CK, being a fundamental cereal, has a beneficial impact on the growth and weight gain of herbivorous animal (Hong et al., 2016). CK has gained recognition in recent times as a fodder shrub due to its nutritional composition, which being rich phenolic acids, alkaloids, and various essential and nonessential amino acids (Long et al., 2020). CT are polyphenolic compounds with a high molecular weight, and it can regulate rumen fermentation and reduce enteric CH_4_ emissions (Tiemann et al., 2008; Adejoro et al., 2019). Thus, we hypothesized that *Caragana korshinskii* tannin(CKT) also has the ability to reduce enteric CH_4_ emissions. The present study investigated the mechanisms of CKT affecting the CH_4_ production using the high-throughput sequencing and untargeted metabolomics technology.

## 2 Materials and methods

The experimental animals, designs, and animal management in the present study were performed in accordance with the National Standard Guidelines for Ethical Review of Animal Welfare (GB/T 35892-2019).

### 2.1 Experimental animals, diets, and design

The experiment was carried out on the experimental farm of the Inner Mongolia Agricultural University, Hohhot, China. Dietary treatments consisted of 500 g/kg concentrate and 500 g/kg roughage, on a DM basis, composed of a variety of forage sources supplied in various quantities. The employed roughage sources were Chinese wildrye, alfalfa and CK. Whole-crop of CK at 60 days of growth were harvested, they were chopped and dried in the shade for 7 days, and dried CK were stored and protected from light until used. CK were used as sources of CT, and CT were measured using the butanol-HCL-method (Makkar, 2003). Table 1 provides information on the chemical composition and CT concentration in CK. A total of 15 crossbred sheep of the Dumont breed, with an average age of 1.5 years and an initial body weight of 40 ± 3 kg, each group consisted of five sheep. The sheep received one of the following feed regimens according to the treatment groups: (1) 400 g/kg concentrate +450 g/kg Chinese wildrye +150 g/kg alfalfa (CON); (2) 400 g/kg concentrate +150 g/kg Chinese wildrye +250 g/kg alfalfa +200 g/kg CK (2% CKT); (3) 400 g/kg concentrate +40 g/kg Chinese wildrye +160 g/kg alfalfa +400 g/kg CK (4% CKT); The three above dietary of sheep treatments had similar energy and CP levels (Table 2). The sheep were housed in the same barn and reared in individual tie stalls (1.5 × 2.5 m) with individual feed trough, the sheep were able to have direct visual and tactile contact and the animals were checked daily by trained personnel to verify the absence of symptoms of disease. Each sheep were given *ad libitum* to clean drinking water in a plastic bucket throughout the whole experiment. The experimental diet was given twice daily at 07:00 and 17:00. Table 2 displayed the components and nutritional content of the diet. The study spanned a total of 74 days, with a 14-day period dedicated to adaptation followed by 60 days of conducting treatments.

**Table 1:** Nutrient composition and CT content in *Caragana korshinskii* (%, air-dry basis).

Items	Content
Dry matter (DM)	95.23
Organic matter (OM)	84.31
Crude protein (CP)	8.75
Ether extract (EE)	0.82
Calcium (Ca)	1.36
Phosphorus (P)	0.04
Neutral detergent fiber (NDF)	65.79
Condensed tannins (CT)	10.78

**Table 2:** Ingredients and nutrient composition of the experimental diet (%, air-dry basis).

Items	CON	2% CKT	4% CKT
Ingredients			
Corn	12.85	21.35	31.35
Wheat bran	18.00	13.00	0.00
Soybean meal	6.00	3.00	6.00
Chinese wildrye	45.00	15.00	4.00
Alfalfa	15.00	25.00	16.00
*Caragana korshinskii*	0.00	20.00	40.00
Limestone	0.50	0.00	0.00
CaHPO_4_	1.00	1.00	1.00
NaCl	0.65	0.65	0.65
Premix^1^	1.00	1.00	1.00
Nutrition level			
Metabolizable energy, (MJ/kg)^2^	8.50	8.42	8.34
CP	10.67	10.71	10.75
NDF	40.36	42.27	44.05
Non-structure carbohydrate (NSC)	36.00	34.49	33.06
Starch	19.85	22.77	22.00
Ca	0.79	0.84	0.78
P	0.53	0.47	0.56

1 The premix provided the following per kilogram of diet: Cu 25 mg, Fe 55 mg, Mn 25 mg, Zn 116 mg, I5.1 mg, Se 0.3 mg, VA 18,000 IU, VD 3,000 IU, VE 576 IU.

2 ME was calculated by the equations from NRC (2001).

### 2.2 Chemical analysis

The feed samples were sent to the Inner Mongolia Key Laboratory of Animal Nutrition and Feed Science, College of Animal Science, Inner Mongolia Agricultural University, for DM and chemical analysis. To determine the DM content, the samples were oven-dried at 65°C until a constant weight was achieved. Upon drying, the samples were through a 1-mm screen. The ground samples were analyzed for ashes after 4 h of combustion in a muffle furnace at 550°C [Association of Official Analytical Chemists, 1990: method 942.05]. The DM, ash and ether extract (EE) of feed and feces samples were analyzed according to Association of Official Analytical Chemists, (1990) using the methods No. 934.01, 920.39, and 924.05, respectively. The OM of the samples was calculated by DM minus ash. The NDF and ADF were analyzed on an Ankom A200i Fiber Analyser (Ankom Technology Co., New York, NY, United States) using the methods of Damiano et al. (2023). CP using a Kjeldahl nitrogen analyzer [Association of Official Analytical Chemists, 1990: method 984.14]; Starch [Association of Official Analytical Chemists, 1990: method 996.11]; Calcium (Ca) [Association of Official Analytical Chemists, 1990: method 927.02]; Phosphorus (P) [Association of Official Analytical Chemists, 1990: method 965.17].

### 2.3 Digestibility trial

The trial to determine digestibility was carried out one week prior to the conclusion of the experiment. Feces from each sheep were collected daily using a plastic screen and weighted prior to being fed. Before determining chemical composition, the feed and feces samples were oven-dried at 65°C for 48 h. Then, determining DM, CP and ash according to Association of Official Analytical Chemists, (1990). The OM was determined by deducting the ash content from the DM of the samples. The determination of NDF and ADF was conducted using the approach outlined by Damiano et al. (2023). According to Chen et al. (2015), nutrient apparent digestibility = (total nutrients intake - nutrients in feces)/total nutrients intake.

### 2.4 Rumen fermentation index measurement

Samples of rumen fluid were obtained at 6 h after morning feeding on the final day of the experiment through utilizing a rumen tube with vacuum pump. To prevent contamination from saliva, the initial 5 mL of rumen fluid were discarded. The pH value of rumen was immediately measured after collection. Subsequently, rumen fluid sample underwent filtration using 4 layers of cheesecloth to analysis volatile fatty acids (VFA), ammonia nitrogen (NH3-N), ruminal methanogens, and metabolites. The filtered sample was divided into 4 parts and frozen (−20°C) for further analysis.

Gas chromatography (GC-2014, Shimadzu International Trading CO. LTD) was used to detect the total concentrations of VFA, following the method outlined in Liu et al. (2019). 15-mL rumen fluid from each sheep was centrifuged (10,000 × g) for 10 min. Following this, 4 mL of the resulting liquid was moved to a centrifuge tube that already contained 1 mL of metaphosphoric acid with a concentration of 25%. After being held for a duration of 30 min and then centrifuged (10,000 × g) for 15 min. For gas chromatographic determination, a sample bottle was filled with a 1.5-mL supernatant using a pipette.

Each sheep’s rumen fluid sample, measuring 10 mL, underwent centrifugation at 10,000 × g for 10 min. Then, 0.5 mL of the resulting supernatant was combined with 4.5 mL of hydrochloric acid (0.2 mol/L) to determine NH_3_-N concentrations, following the method outlined in Pellikaan et al. (2011).

### 2.5 Methane production

The open-circuit respirometry system with 6 metabolism cages (Sable Systems International, NV, United States) was utilized to measure CH_4_ production. A total of 15 sheep were split into three groups for determination. After a 48-h adaptation period, there was a subsequent 48-h duration dedicated to measuring methane emissions, The specifics regarding its explanation, functioning, and calibration are given in Salazar et al. (2018). The net CH_4_ concentration was determined by subtracting the ambient-air CH_4_ concentration from the CH_4_ concentration of air released from each chamber. To calculate the overall daily CH_4_ emission, the net CH_4_ concentration for each chamber was multiplied by each chamber’s daily air volume.

### 2.6 Analysis of methanogens community

The DNA Kit (Omega Bio-tek, Norcross, GA, United States) was used to extract the total DNA of sample. The NanoDrop2000 (Thermo Fisher, NY, United States) was used to detect the purity and concentration of DNA. A universal primer was used to amplify the 16S rRNA gene’s V3-V4 region 338F (5’-GGTGGTGTMGGATTCACACARTAYGCWACAGC-3′) and 806R (5’-TTCATTGCRTAGTTWGGRTAGTT-3′), with microbial DNA serving as the template. The data were processed and analyzed on the Majorbio cloud platform.^1^

### 2.7 Non-targeted metabolomics analysis

#### 2.7.1 Gas chromatography–mass spectrometry metabolomics analysis

Metabolomics analysis was conducted using the fluid from the rumen. A 200 μL sample of rumen fluid was moved into a centrifuge tube with a capacity of 1.5 mL, and then 300 μL of extraction solution (a mixture of methanol and acetonitrile in a ratio of 2:1) was introduced. After vortexing the mixture for 30 s, it was subsequently centrifuged at a speed of 13,000 revolutions per minute for 15 min at a temperature of 4°C. Following centrifugation, the liquid above the sediment was subjected to drying in a vacuum concentrator. Following the process of evaporation, the sample was supplemented with 80 μL of hydrochloride of methoxy amination (15 mg/mL in pyridine) and left to incubate for 90 min at a temperature of 37°C. The blend was placed in an incubator at a temperature of 70°C for a duration of 60 min. The samples that underwent derivatization were examined utilizing the Agilent 8890B-5977B gas chromatograph system manufactured by Agilent, located in Santa Clara, CA, United States.

#### 2.7.2 Metabolomics data processing and analysis

MassHunter workstation Quantitative Analysis (v10.0.707.0) software was used to process the initial data. The preprocessed data were analyzed via Majorbio Cloud Platform. PCA and OPLS-DA were conducted using the R program’s ropls package (Version 1.6.2). The FC was utilized to assess the alteration pattern (upregulation or downregulation) of distinct metabolites. Significantly differential metabolites were chosen based on the variable important in projection (VIP) acquired from the OPLS-DA model and the *p*-value derived from Student’s *t*-test. Metabolites that had a VIP score greater than 1.5 and a *p*-value less than 0.05 were determined to be the metabolites that showed significant differences among the three groups. The metabolite distance algorithm was Bray-Curtis. The KEGG database was used to annotate the metabolic pathways where the distinct metabolites were grouped. Pathway enrichment analysis was conducted using the Python software (version 1.0.0) as described by Artegoitia et al. (2017). To investigate the metabolites essential for distinguishing between groups, we employed Receiver Operator Characteristic (ROC) analysis using SPSS (version 22) Statistics. As the area under curve (AUC) value approached 1, the prediction accuracy increased.

### 2.8 Correlation analysis

The correlation analysis was performed utilizing the tools provided by OmicShare. Spearman’s correlation coefficient was used to calculate the associations between distinct *methanogens* and metabolites, rumen fermentation parameters, and CH_4_ production. Additionally, the correlation coefficient was employed to determine the significant relationships between specific metabolites and CH_4_ production. The correlation coefficient (*r*) ranged from −1 to 1, with positive correlation represented by *r* > 0 and negative correlation represented by *r* < 0.

### 2.9 Statistical analysis

In order to determine the effect of treatments on the nutrient apparent digestibility, the alpha diversity indexes, rumen fermentation parameters, and CH_4_ production, in the present study, each sheep was considered as an experiment unit. The normality of the distribution of variables was tested by the Shapiro - Wilk test and all variables were normally distributed. All results of 3 dietary treatment groups were subjected to ANOVA where diets were treated as the fixed effect, period, and animal were considered as the random effect using the GLM procedure of SAS (SAS Inst. Inc., Cary, NC, USA; version 9.0). When significant differences were observed, Tukey’s test was used to adjust for multiple comparisons. The PROC MIXED model was used including random and fixed effects as follows: *Y_ij_* = μ + *L_i_* + *T_j_* + *ε_ij_*, where *Y_ij_* is the dependent variable, μ is the overall mean, *L_i_* is the random effects of sheep (*i* = 5), *T_j_* is the fixed effect of CKT supplements (*j* = 0, 2 and 4%), and *ε_ij_* is the error term. Variability in the data was expressed as the standard error means and a probability level of *p* < 0.05 was considered statistically significant.

## 3 Results

### 3.1 The effect of CKT on rumen fermentation characteristics

The rumen fermentation parameters were listed in Table 3. In comparison to the control group, the 2 and 4% CKT groups exhibited a significant decrease (*p* < 0.05) in NH_3_-N and acetate concentrations. Rumen fluid pH, total VFA (TVFA), propionate, butyrate and valetate concentration and acetate: propionate (A/P) were not altered (*p* > 0.05) by addition of CKT.

**Table 3:** Effect of CKT on rumen fermentation parameters in sheep.

Items	CON	2% CKT	4% CKT	SEM	*p*-value
pH	6.41	6.57	6.33	0.119	0.368
NH_3_-N, mg/100 mL	18.17^a^	14.31^b^	12.86^b^	0.814	0.002
TVFA, mmol/L	91.64	82.90	82.04	3.390	0.155
Acetate, mmol/L	60.98^a^	53.33^b^	52.67^b^	1.859	0.022
Propionate, mmol/L	17.73	15.94	13.48	0.989	0.139
Butyrate, mmol/L	14.73	13.26	12.83	0.487	0.117
Valetate, mmol/L	1.40	1.17	1.34	0.084	0.181
A/P	3.583	3.663	3.061	0.296	0.607

CON = control group; CKT = *caragana korshinskii* tannin; NH_3_-N = ammonia nitrogen; BCP = bacteria protein; A/P = acetate: propionate ratio; VFA = volatile fatty acids; ^a, b^ within a row, different letters mean differed significantly (*p* < 0.05); SEM = standard error of mean.

### 3.2 The effect of CKT on intake and nutrient apparent digestibility

As shown in Table 4, dietary treatments did not show any effect on DM, OM, CP, NDF and ADF intake of animal. Similarly, the addition of 2% CKT did not have impact (*p* > 0.05) on the apparent digestibility of NDF. However, the addition of 4% CKT significantly increased (*p* < 0.05) the apparent digestibility of NDF. The addition of CKT at 2 and 4% resulted in a significant reduction (*p* < 0.05) in the apparent digestibility of CP. Both 2 and 4% CKT had no impact (*p* > 0.05) on the apparent digestibility of DM, OM, ADF and the intake of DM, OM, CP, NDF, ADF.

**Table 4:** Effect of CKT on feed intake and nutrient apparent digestibility in sheep.

Items	CON	2% CKT	4% CKT	SEM	*p*-value
Intake (kg/d)					
DM	1.53	1.63	1.45	0.105	0.608
OM	1.49	1.59	1.41	0.010	0.581
CP	0.16	0.17	0.15	0.001	0.623
NDF	0.62	0.69	0.64	0.044	0.618
ADF	0.28	0.29	0.24	0.018	0.2037
Nutrient apparent digestibility (%)					
DM	55.07	55.04	55.84	0.022	0.959
OM	57.56	57.25	60.82	0.020	0.429
CP	64.86^a^	49.75^c^	56.30^b^	0.010	0.006
NDF	49.70^b^	48.85^b^	62.55^a^	0.019	0.002
ADF	60.44	53.28	60.69	0.023	0.276

CON = control group; CKT = *Caragana Korshinskii* tannin; DM = dry matter; OM = organic matter; CP = crude protein; NDF = neutral detergent fiber; ADF = acid detergent fiber; ^a, b^ within a row, different letters mean differed significantly (*p* < 0.05); SEM = standard error of mean.

### 3.3 The effect of CKT on CH4 emission

Table 5 shows that the inclusion of 2 and 4% CKT resulted in a significant reduction (*p* < 0.05) in daily CH_4_ emission (L CH_4_/d). CH_4_ production of DMI, OMI, NDFI and metabolic weight (BW^0.75^) did not differ significantly (*p* > 0.05) among the various treatments.

**Table 5:** Effect of CKT on CH_4_ emission in sheep.

Items	CON	2% CKT	4% CKT	SEM	*p*-value
L CH_4_/day	15.6^a^	10.62^b^	11.94^b^	0.320	<0.001
L CH_4_/kg of DMI	11.29	9.56	10.81	0.664	0.333
L CH_4_/kg of OMI	12.53	11.87	12.14	1.144	0.952
L CH_4_/kg of NDFI	26.44	23.70	24.87	2.314	0.817
L CH_4_/kg of BW^0.75^	0.80	0.74	0.74	3.314	0.870

CON = control group; CKT = *Caragana korshinskii* tannin; BW^0.75^ = body weight raised to the power 0.75; ^a, b^ within a row, different letters mean differed significantly (*p* < 0.05); SEM = standard error of mean.

### 3.4 The effect of CKT on the richness, diversity, and composition of ruminal methanogens

A total of 193,249 effective 16S rRNA sequences were obtained from 15 rumen fluid samples and 97 OTU were obtained by performing OTU clustering on nonrepetitive sequences according to 97% similarity. The analysis of α-diversity indicated a decrease in the Chao index in the 4% CKT group (*p* < 0.05), and the ACE index for both the 2 and 4% CKT groups showed a significant decrease (*p* < 0.05) compared to the CON group. Meanwhile, a significant increase(*p* < 0.05) in the Simpson index was observed in the 4% CKT group. Which demonstrated that adding CKT decreased the diversity and richness of ruminal methanogen communities (Table 6).

**Table 6:** Effect of CKT on α-diversity of ruminal methanogens in sheep.

Items	CON	2% CKT	4% CKT	SEM	*p*-value
Shannon	1.19	1.52	1.57	0.122	0.433
Simpson	0.22^b^	0.31^ab^	0.39^a^	0.053	0.018
ACE	39.90^a^	35.48^b^	35.29^b^	1.451	0.005
Chao	45.44^a^	39.31^a^	34.33^b^	3.214	0.005

CON = control group; CKT = *caragana korshinskii* tannin; ^a, b^ within a row, different letters mean differed significantly (*p* < 0.05); SEM = standard error of mean.

### 3.5 The significantly different ruminal methanogens between the control and CKT groups

**Figure 1:**
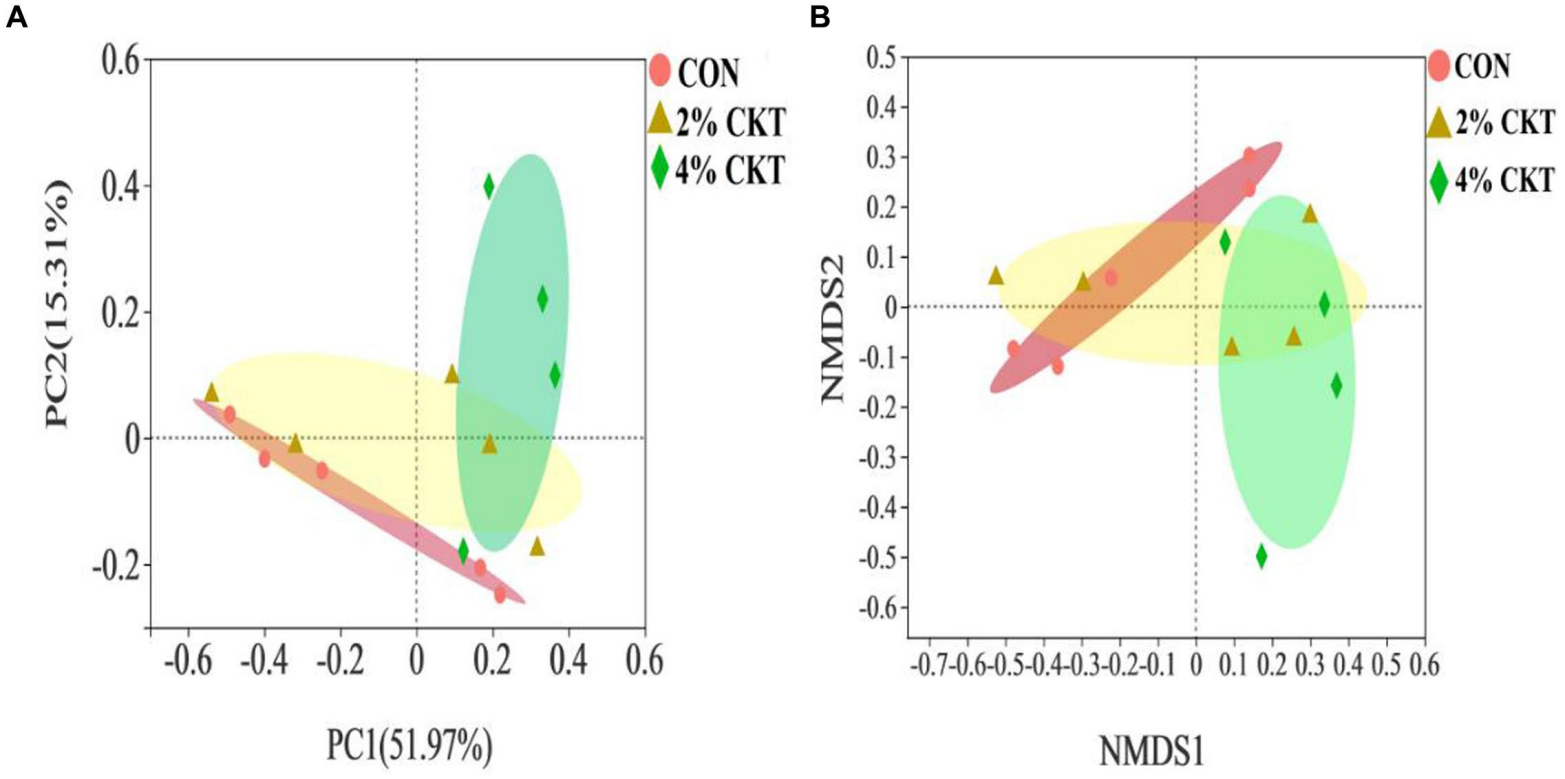
Beta diversity analysis of ruminal through **(A)** principal coordinate analysis (PCoA) and **(B)** non-metric multidimensional scaling analysis (NMDS). CON, control group. CKT, *caragana korshinskii* tannin; PC = principal components.

The β-diversity analysis was performed to explore differences in the rumen *methanogens* community between the treatment groups (Figure 1). The PCoA and NMDS plots, using the Bray-Curtis distance matrix, revealed a clear separation between the rumen *methanogens* in the control group and the 4% CKT group. Different quadrants of the coordinate axis held these points, indicating the intake of 4% CKT had a noticeable impact on both the species and abundance of rumen *methanogens*.

At the phylum level, norank_d_Bacteria (34.62, 37.50 and 53.48%) and *Euryarchaeota* (52.12, 47.83 and 18.86%) were the dominant microbe in the control, 2% CKT and 4% CKT groups, respectively. The relative abundance of norank_d_Bacteria was increased in the 4% CKT compared with the CON and 2% CKT groups (*p* < 0.05). In contrast, *Euryarchaeota* was decreased in rumen with the 4% CKT addition (*p* < 0.05). At the genus level, norank_d_Bacteria (34.62, 37.50 and 53.48%), *Methanobrevibacter* (50.61, 45.76, and 16.60%), unclassified_d_Unclassified (13.26, 14.67, and 27.65%) and *Methanosphaera* (0.86, 1.38, and 1.63%) were the predominant genera in the control, 2% CKT and 4% CKT groups, respectively. The relative abundance of norank_d_Bacteria and unclassified_d_Unclassified were increased in the 4% CKT compared with the CON and 2% CKT groups (*p* < 0.05). In contrast, *Methanobrevibacter* was decreased in rumen with the 4% CKT addition (*p* < 0.05) (Table 7).

**Table 7:** Effect of CKT on species proportion of rumen methanogensin sheep.

Items	CON	2% CKT	4% CKT	SEM	*p*-value
Phyla level					
norank_d_Bacteria	34.62^b^	37.50^b^	53.48^a^	0.090	<0.001
Euryarchaeota	52.12^a^	47.83^a^	18.86^b^	0.128	<0.001
others	13.26^b^	14.68^b^	27.65^a^	0.070	<0.001
Genus level					
norank_g_Bacteria	34.62^b^	37.50^b^	53.48^a^	0.090	<0.001
Methanobrevibacter	50.61^a^	45.76^a^	16.60^b^	0.134	<0.001
unclassified_d_Unclassified	13.26^b^	14.67^b^	27.65^a^	0.075	0.009
Methanosphaera	0.86	1.38	1.63	0.007	0.177
unclassified_c_*Thermoplasmata*	0.26	0.32	0.49	0.001	0.099
others	0.39^a^	0.37^a^	0.15^b^	0.001	0.006

CON = control group; CKT = *caragana korshinskii* tannin; ^a, b^ within a row, different letters mean differed significantly (*p* < 0.05); SEM = standard error of mean.

### 3.6 The effect of CKT on ruminal metabolites in sheep

**Figure 2:**
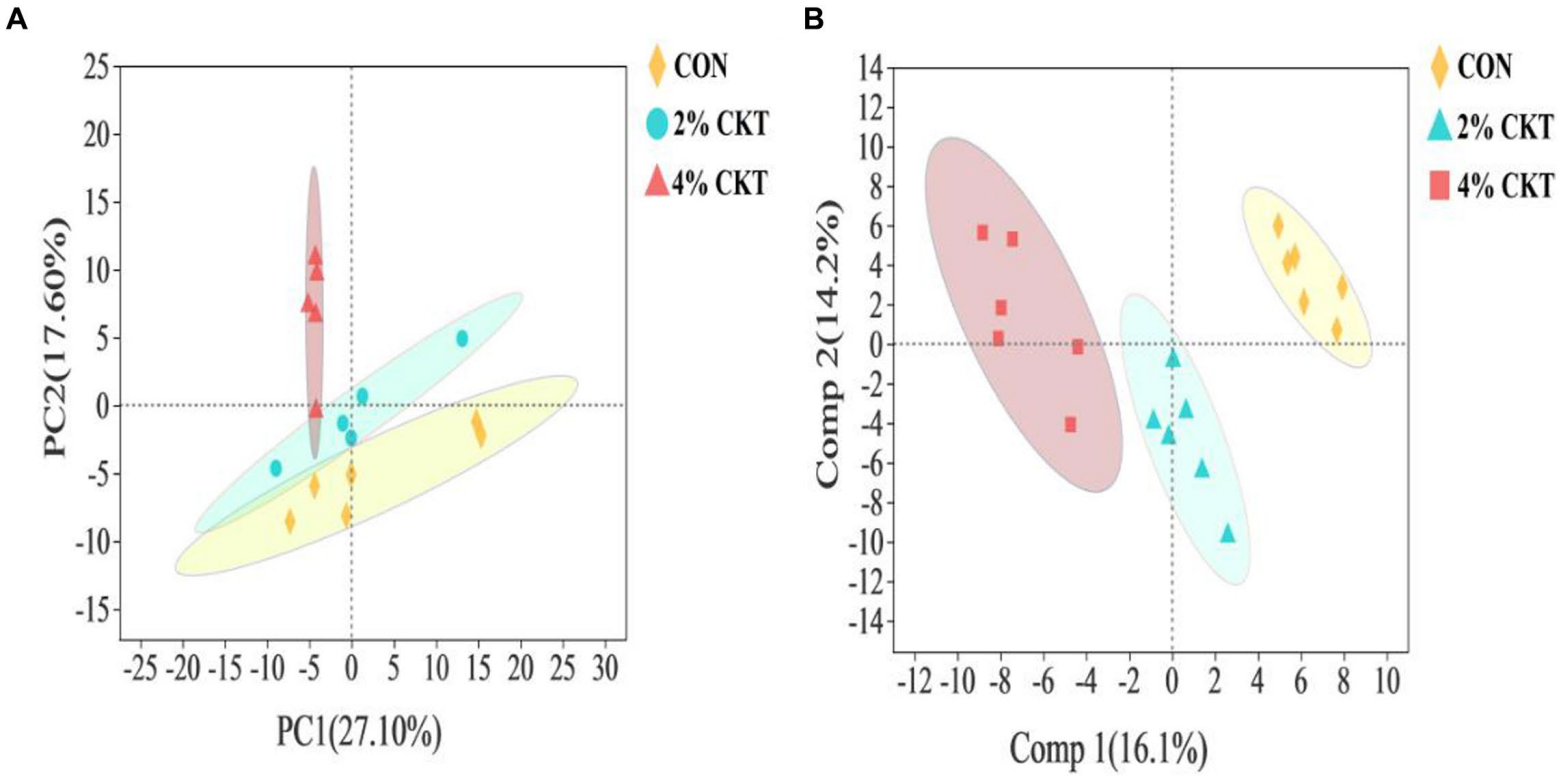
The principal component analysis (PCA) score plot **(A)** and orthogonal partial least squares discriminant analysis (OPLS-DA) score plot **(B)** of rumen metabolites. CON, control group; CKT = *Caragana korshinskii* tannin.

Untargeted metabolomics techniques were used to analyze the rumen metabolites, resulting in the identification of a grand total of 261 metabolites. The PCA analysis, which is a type of unsupervised multivariate statistical analysis, indicated that there was a clear distinction in the ruminal metabolites between the sheep in the CON and 4% CKT groups (Figure 2A). The variations in metabolites were additionally confirmed through the score plots of OPLS-DA (Figure 2B). The stability and reliability of the OPLS-DA model are confirmed by the cumulative values of R^2^Y (0.786) and Q^2^ (0.530) in the OPLS-DA plot.

### 3.7 The significantly different ruminal metabolites between the control and 4% CKT groups

In Table 8, it was found that there were 25 distinct metabolites in the sheep’s rumen that differed between the control group and the 4% CKT group, with VIP scores exceeding 1.5 and *p*-values less than 0.05. Two clusters were formed to distinguish the differential metabolites between the two groups. The relative expression of dibenzofuran, heptanoic acid, 4-hydroxypyridine, N-methy-L-glutamic acid, N-carbobenzoxy-L-leucine, 1-octanol, N-alpha-acetyl-L-lysine, 2,3-butanediol and apigenin were elevated in the rumen of sheep fed 4% CKT compared with the CON group. However, The relative expression of tyramine, cyclohexanecarboxylic acid, 5-hydroxyindole-2-carboxylic acid, 2,6-diaminopimelic acid, asarylaldehyde, 4-O-methylphloracetophenone and 3,7-dihydroxyflavone etc. were higher in the CON group than that of 4% CKT group.

**Table 8:** Significant differential metabolites between the sheep of CON and 4% CKT groups (VIP > 1.5; *p* < 0.05).

Metabolite	RT	VIP	*p*-value	FC
dibenzofuran	5.7270	1.6348	1.96 × 10^−5^	1.07
heptanoic acid	21.4349	1.6312	0.0084	1.08
4-hydroxyquinoline	21.7530	1.7839	2.35 × 10^−5^	1.09
N-methy-L-glutamic acid	9.6117	1.6028	0.0042	1.07
N-carbobenzoxy-L-leucine	10.3013	1.8354	7.31 × 10^−4^	1.08
1-octanol	15.6110	2.4413	1.57 × 10^−8^	1.17
N-alpha-acetyl-L-lysine	17.6510	1.8719	0.0028	1.11
2,3-butanediol	20.8617	2.6669	5.26 × 10^−9^	1.13
Apigenin	22.0149	1.9480	2.90 × 10^−4^	1.10
tyramine	6.6129	1.6755	0.0241	0.94
cyclohexanecarboxylic acid	8.6991	4.315	5.93 × 10^−13^	0.63
5-hydroxyindole-2-carboxylic acid	12.5662	2.175	0.0170	0.82
2,6-diaminopimelic acid	15.1418	1.6772	0.0206	0.90
N-acetyl-5-hydroxytryptamine	16.6542	1.9410	6.60 × 10^−6^	0.91
2,3-dihydroxybenzoic acid	17.0901	1.9703	4.98 × 10^−5^	0.92
uric acid	18.5556	1.9084	0.0020	0.89
urea	20.4142	2.1341	0.0071	0.90
3-methyl-L-histidine	20.0321	2.6197	2.78 × 10^−9^	0.84
D-saccharic acid	21.1464	1.6698	0.0101	0.91
mandelic acid	24.7449	1.5584	9.11 × 10^−8^	0.95
Asarylaldehyde	24.9037	1.5284	0.0240	0.91
3,7-Dihydroxyflavone	26.5155	1.9023	0.0061	0.89
tyrosine methyl ester	26.5221	1.8031	7.61 × 10^−8^	0.92
4-O-Methylphloracetophenone	30.4996	3.0017	1.00 × 10^−8^	0.78
2-ethyltoluene	35.6959	1.6474	1.91 × 10^−4^	0.93

RT, Retention time; VIP, variable importance in the projection; FC, fold change, FC > 1 means that this metabolite is higher in 4% CKT group than that in CON group and FC < 1 means that this metabolite is lower in 4% CKT group.

**Figure 3:**
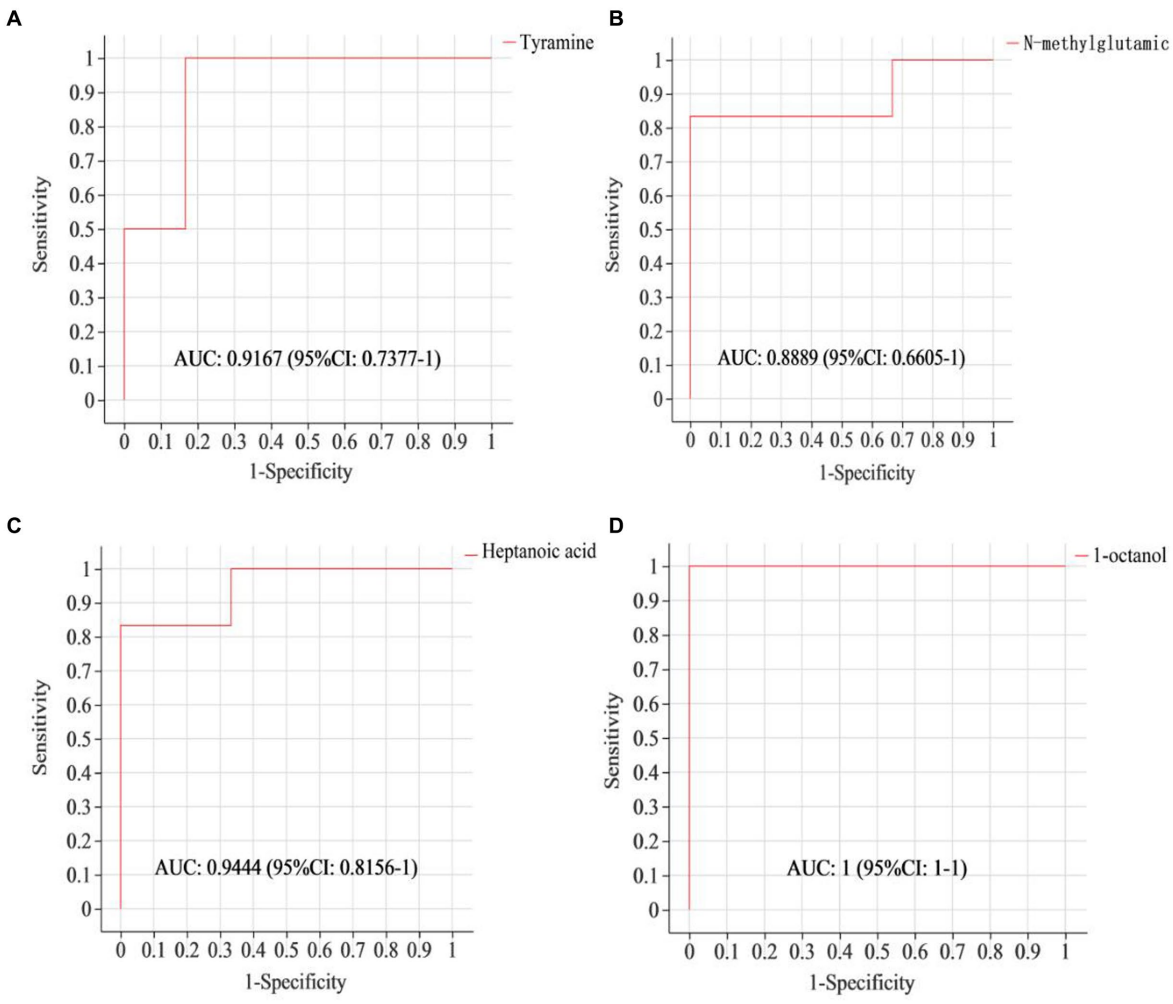
ROC curves to evaluate the differential metabolites that have key impact on the differentiation between the sheep of CON and 4% CKT groups. ROC curve reflects the relationship between sensitivity and specificity. The *x*-axis is specificity (false positive rate). The closer the x-axis is to zero, the higher the accuracy will be. The *y*-axis is sensitivity (true positive rate). The larger the y-axis is, the better the accuracy is. The AUC is used to indicate the accuracy of prediction. AUC value close to 1 suggested the higher the accuracy of prediction. **(A)** Tyramine; **(B)** N-methylglutamic acid; **(C)** Heptanoic acid; **(D)** 1-octanol.

To assess if the identified differential metabolites were crucial for distinguishing between different groups, a ROC curve was generated. As shown in Figure 3, the AUC of tyramine, N-methylglutamic acid, heptanoic acid and 1-octanol were all above 0.85, demonstrating that these specific ruminal metabolites effectively indicate the impact of a 4% CKT diet on rumen metabolism.

### 3.8 Metabolic pathway enrichment analysis of differentially abundant metabolites

**Figure 4:**
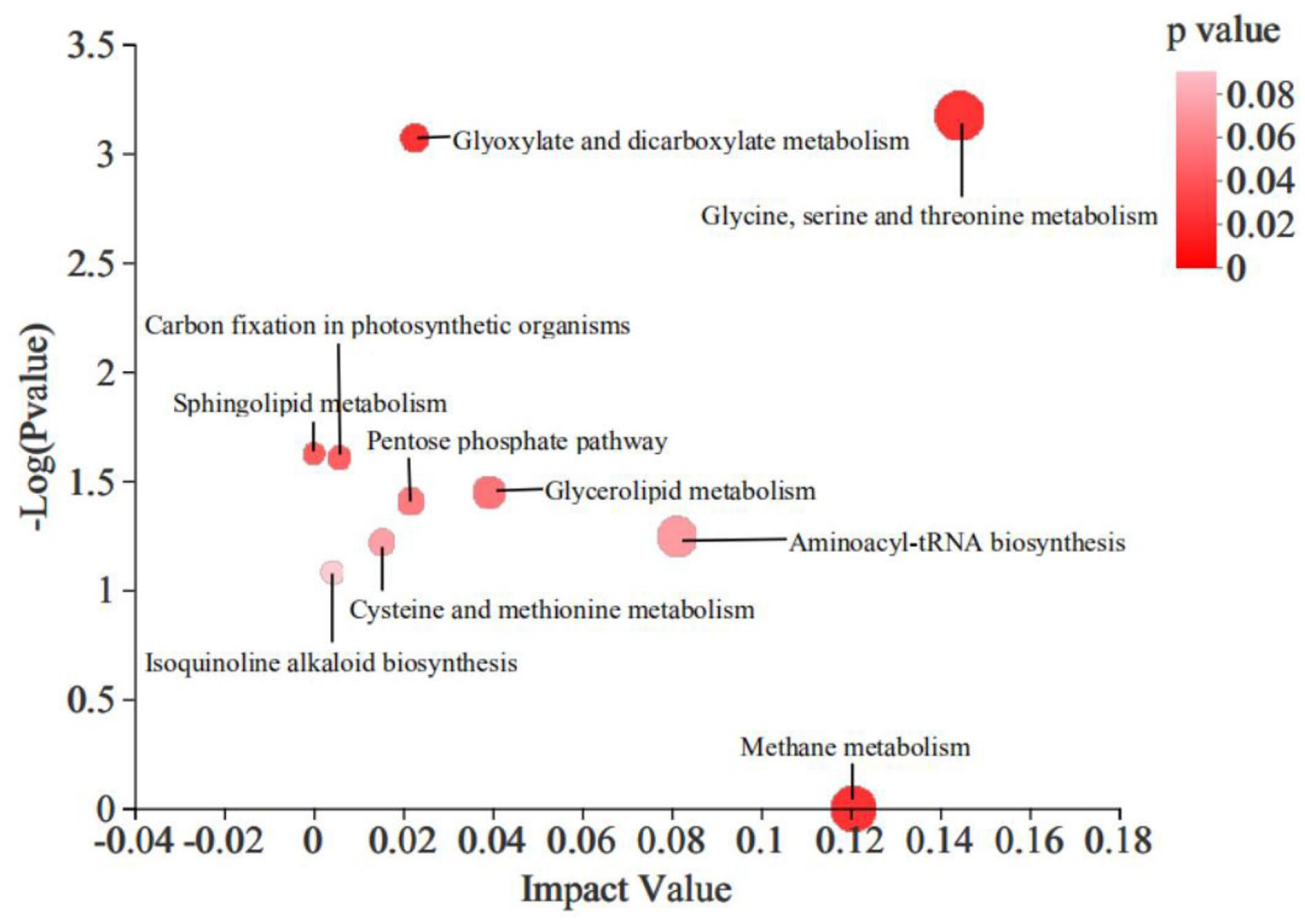
Differential metabolic pathway enrichment analysis of significantly differential metabolites in rumen. The large size indicates high pathway enrichment, and dark color indicates high pathway impact values.

The KEGG pathway enrichment analysis revealed that the addition of 4% CKT affected primarily CH_4_ metabolism (*p* < 0.0001), glycine, serine and threonine metabolism (*p* = 0.001), mminoacyl-tRNA biosynthesis (*p* = 0.057), glycerolipid metabolism (*p* = 0.036), glyoxylate and dicarboxylate metabolism (*p* = 0.001), pentose phosphate pathway (*p* = 0.039), cysteine and methionine metabolism (*p* = 0.060), isoquinoline alkaloid biosynthesis (*p* = 0.083), carbon fixation in photosynthetic organisms (*p* = 0.025) and sphingolipid metabolism (*p* = 0.024) in the rumen of sheep (Figure 4).

### 3.9 Correlation analysis among differential ruminal methanogens, metabolites, CH4 production, and rumen fermentation parameters

**Figure 5:**
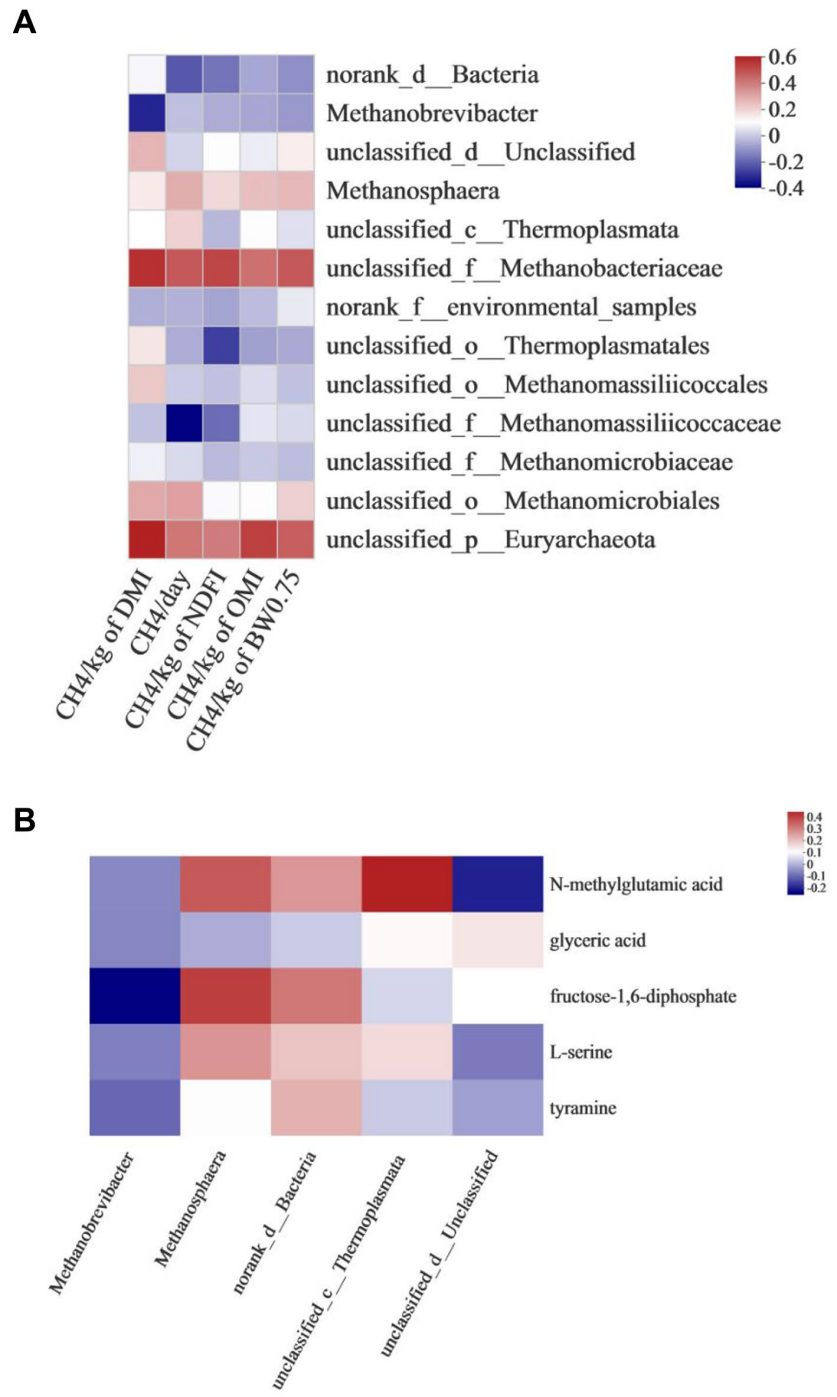
Correlation analysis between **(A)** rumen *methanogens* and CH_4_ production, **(B)** rumen *methanogens* and differential metabolites associated with methane metabolism.

Correlation analysis between rumen *methanogens* and CH_4_ production showed that the emission of CH_4_/kg of DMI was positively associated with unclassified_f_*Methanobacteriaceae* (*r* = 0.550, *p* = 0.034) and unclassified_p_*Euryarchaeota* (*r* = 0.584, *p* = 0.022). CH_4_/day was negatively associated with unclassified_f_*Methanomassiliicoccaceae* (*r* = −0.386, *p* = 0.046). CH_4_/kg of OMI was positively associated with unclassified_p_*Euryarchaeota* (*r* = 0.515, *p* < 0.05) (Figure 5A).

The correlation between rumen *methanogens* and differential metabolites associated with methane metabolism is shown in Figure 5B. M*ethanobrevibacter* was negatively associated with fructose- 1,6 -diphosphate (*r* = −0.4227, *p* = 0.037). *Methanosphaera* was positively associated with fructose- 1,6 -diphosphate (*r* = 0.3675, *p* = 0.018). Unclassified_c_*Thermolplasmata* was positively associated with N-methylglutamic acid (*r* = 0.5095, *p* = 0.029).

## 4 Discussion

### 4.1 CKT affected rumen fermentation, nutrient digestibility, and CH4 emission of sheep

Regarding rumen pH, no differences were found among the sheep, which aligned with the findings of Phesatcha and Wanapat (2017) who reported that average pH values of 6.5 ± 0.2 at CT concentration of <2%. Maintaining a stable pH in the rumen is essential for the proliferation of microorganisms and the fermentation of feed (María et al., 2020). Our results further indicated that CKT did not destroy rumen homeostasis.

To be certain to put in evidence any effect on CH_4_ production, various research studies have demonstrated alterations in VFA production and nutrient digestibility when employing quebracho tannins, fumarate, and propionate precursors, further affecting CH_4_ synthesis and metabolism. For instance, there was a decrease in acetate concentration and the A/P ratio when 10 and 20 g/kg DM of quebracho tannin extracts were added to sheep dietary (Beauchemin et al., 2007). This subsequently lowers the quantity of hydrogen available for methanogenesis, as mentioned in a research by Patra and Jyotisna (2009). Study carried out utilizing propionate precursors, like acrylate (Newbold et al., 2005), demonstrated a decrease in CH_4_ yield while observing an increase in acetate and TVFA production. Amlan et al. (2017) found that the decrease in CH_4_ emissions from the digestive system was affected by the digestibility of CP. Similar responses were discovered in this research, where the decrease in intestinal propionate concentration and CH_4_ production was impacted by the digestibility of NDF. Molina-Botero et al. (2019) observed a reduction in the apparent digestibility of NDF by including *E. cyclocarpum* and *G. sepium* (contain CT), this could be explained by the multiple adverse effects of CT’s on animal fermentative processes. CT negatively affected the adhesion of cellulolytic bacteria to the substrate, reduced the activity of fibrolytic enzymes, and inhibited the growth of the cellulolytic population or form complexes with cellulose. However, in the present study, the digestibility of NDF was increased in 4% CKT group, this could be attributed to the different activity and molecular structure of tannins, which needs to be further studied.

NH_3_-N serves as a crucial nitrogen provider for the process of rumen fermentation and the development of microorganisms. Increased availability of NH_3_-N in the rumen results in higher nitrogen loss and stunted growth (Kabasa et al., 2004). In this research, the inclusion of 2 and 4% CKT in the diet resulted in the concentration of NH_3_-N decreased significantly, suggesting a reduction in ruminal CP degradation. Other research studies have also yielded comparable findings when assessing quebracho CT at a daily dosage of 18 g/kg in cows (Aguerre et al., 2016). The decrease in NH_3_-N concentration corresponded to the decrease in CP digestibility in the rumen of sheep that were fed 2 and 4% CKT. The decreased digestibility of CP observed in the 2% CKT and 4% CKT group is associated with the capacity of tannin to attach to protein. Furthermore, it is plausible that a portion of this compound (tannin-proteins) did not separate in the abomasum (Waghorn, 2007). Furthermore, when tannin is released into the duodenum, it has the potential to deactivate enzymes in the intestines or attach itself to proteins (Warren et al., 1998), ultimately leading to a decrease in the digestibility of proteins. Similar results were observed by Tiemann et al. (2008) with black wattle CT, there was a gradual decrease in CP digestibility as the levels of up to 20 g/kg of DM were included in the diets of Jersey steers. Al-Dobaib (2009) reported a negative impact of 15 g kg^−1^ CT from quebracho on protein degradation in an *in vitro* experiment with rumen fluid from sheep. Other studies reported that condensed or hydrolysable tannin concentrations below 15 g/kg had no impact on *in vivo* ruminal protein degradation (Benchaar et al., 2008; Krueger et al., 2010). The inclusion of 2 and 4% CKT resulted in a significant reduction in daily CH_4_ emission can be attributed to the reduced digestibility of CP in the rumen, as observed in current research.

Rumen microorganisms degrade carbohydrates to form VFAs, CO_2_, H_2_, CH_4_, adenosine triphosphate (ATP), and a few other compounds. VFAs account for 70–80% of the energy sources of ruminants (Jayanegara et al., 2011). Vasta et al. (2019) provided a summary of the existing literature on the alterations in TVFA levels and the acids acetic, propionic, butyric, and valeric following tannin usage. Five parameters showed varying levels, ranging from increased to unchanged to decreased. The precise impact of tannin on the alteration of the molar ratios of VFAs in the rumen remains uncertain, and several authors have proposed that differences in tannin structure and activity due to different plant sources and extraction conditions may explain this inconsistency (Patra et al., 2017). Hence, it is crucial to examine the impacts of tannin inclusion on a per-instance basis. The absence of disparities in TVFA, propionic, butyric, and valeric acids in the present research might be attributed to the restriction of animal numbers per treatment, which minimized the variability linked to the impacts of diet or the insignificant impact of dietary additives on VFA absorption through the rumen wall (Bodas et al., 2012). These results align with previous studies (Aguerre et al., 2016; Piñeiro-Vázquez et al., 2018). The decrease in acetate levels could be associated with the impact of CT on the breakdown of fiber in the rumen. According to Castro-Montoya et al. (2011), cellulolytic bacteria exhibit greater susceptibility to elevated levels of CT in comparison to other microorganisms. These substances not only create complexes with plant cell wall carbohydrates but also directly hinder the activity of CT in these microorganisms. The CT caused a shift in the usage of carbon chain for the creation of VFAs in the rumen, resulting in a reduction in the concentration of acetic acid. This alteration in rumen fermentation could potentially lead to a decreased amount of free metabolic hydrogen, ultimately resulting in limited availability for CH_4_ production (Hassanat and Benchaar, 2013). Which was another reason for CH_4_ emission reduction effect of CKT.

### 4.2 Mechanisms of CKT decreasing CH4 emission in rumen of sheep

In present work, we confirm the strong effect of depression of CKT on methanogensin population. In addition, as above mentioned, the mechanisms in which CKT reduce CH_4_ emission have two mechanisms, i.e., (1) through reduction in CP digestibility and increase in NDF digestibility, and (2) through decrease concentration of acetic acid, this change in the rumen fermentation may have led to a decrease in the amount of free metabolic hydrogen available for CH_4_ production. Nevertheless, Cammack et al. (2018) reported that microorganisms had a significant impact on the effective functioning of the rumen. Protozoa utilize starch, cellulose, hemicellulose, pectin, and soluble sugars to generate VFA and H_2_. The *methanogens* that are attached to the surface of protozoa employ available H_2_ to produce CH_4_, thereby reduce the negative pressure in the rumen (Caridad et al., 2012). In the absence of *methanogens*, the levels of methane can be reduced by approximately 11% (Newbold et al., 2015). Thus, high-throughput sequencing technology was used to detect the population diversity of *methanogens*in the current study, results showed that the relative abundance of *Euryarchaeota*, which contained most of the *methanogens*, was lower in the 4% CKT group than that of CON and 2% CKT groups. In addition, the relative abundance of *Methanobrevibacter,* as the prominent methanogen, also was lower in the 4% CKT group than that of the CON and 2% CKT groups. In this sense, several studies provide support for our findings through various studies conducted under *in vitro* conditions using cattle rumen liquor, for example, Tan et al. (2011) observed that the total *methanogens*, as well as *methanogens* belonging to the *Methanobacteriales*, showed a significant decline when CT inclusions exceeded 1 mg/50 mg DM. Meanwhile, Animut et al. (2007a) also noted that goats fed diets containing CT had decreased populations of *methanogens* in their rumen. Liu et al. (2011) observed the phenomenon *in vivo*, the addition of 30 g of CT/kg of diet from chestnut decreased the populations of *methanogens* in sheep. One potential explanation for the antimethanogenic impact is the suppressive influence of tannins on the functions of enzymes essential for the development of *methanogens* (Patra and Saxena, 2011). In addition, several studies (Animut et al., 2007b; Bhatta et al., 2009) found that CT had antiprotozoal properties. As we know, *methanogens* attached to the surface of ciliate protozoa and symbiosed with them (Finlay et al., 1994). Hence, a decline in protozoa populations could potentially lead to a decrease in *methanogen* populations. However, the underlying mechanism behind this process needs further investigation.

The use of metabolomics analysis offers a chance to comprehensively assess a wide range of small molecule metabolites in cells, tissues, and biofluids. While the complete description of the majority of differential rumen metabolites identified in the research is still lacking, the metabolomics findings also indicate that CKT supplementation has a significant impact on methane metabolism in sheep. Tyramine is a monoamine compound derived from the tyrosine, which can be converted to pyruvate under the action of tyrosine aminotransferase (Zhang et al., 2019). The H_2_ produced by the catabolism of pyruvate is the raw material for methane synthesis (Min et al., 2020), thus, the decrease in tyramine levels was consistent with the decrease in the daily CH_4_ emission. Therefore, the decreased levels of tyramine might imply the potential of CKT to downregulate methane synthesis in sheep. Studies have shown that animals can produce formaldehyde through physiological and biochemical reactions and metabolic reactions, which is an important intermediate metabolite (Ma et al., 2020). Through substitution reaction, methane can be converted into methanol, and formaldehyde is the oxidized substance of methanol. N-methy-L-glutamic acid is analog of L-glutamic acid, which can produce formaldehyde through a series of metabolic pathways (Guido et al., 2018). In the present research, the rice in N-methy-L-glutamic acid levels implies that the transition from methane to formaldehyde may be inhibited. The negative correlation between N-methy-L-glutamic acid and *Methanobrevibacter* could be accounted for. The above-mentioned compounds are significant candidates for future investigations due to their reaction to CKT supplementation. Further investigation is warranted to explore the metabolic processes and action mechanism of CKT in the sheep rumen.

However, some limitations need to be acknowledged. First of all, it is obvious that supplementing tannin with CK instead of green hay is not appropriate, since tannin is only one of the main active ingredients in CK, further extraction and purification of this compound is needed to carry out further research. In addition, this preliminary model was developed on a single experimental group at one farm (crossbred sheep of the Dumont breed). Therefore, its generalizability remains to be assessed in multi-farm studies and meta-analyses and is yet to be externally validated. Recognizing these limitations is crucial to properly contextualize the study and the research questions that need to be investigated in order to gain a more comprehensive understanding of the impact of CKT on CH_4_ emission of sheep.

## 5 Conclusion

The results of this experiment demonstrate that the levels of NH_3_-N and acetate in rumen of sheep were reduced when fed 2 and 4% CKT; The digestibility of CP in sheep from 2 and 4% CKT groups was decreased; while the digestibility of NDF was increased in 4% CKT group. Furthermore, the supplementation of CKT resulted in a decrease in daily CH_4_ emissions from sheep by reducing the richness and diversity of ruminal methanogens community, meanwhile decreasing concentrations of tyramine and increasing concentrations of N-methy-L-glutamic acid. However, CH_4_ production of DMI, OMI, NDFI and metabolic weight did not differ significantly across the various treatments. Whether this is related to adaptative mechanisms involved in feed conversion efficiency and rumen digestion process needs further studies preferable under house feeding situations. Collectively, Dietary supplementation with 4% CKT/kg DM per sheep could reduce CH_4_ emission through reduction in CP digestibility, increase in NDF digestibility and decrease concentration of acetic acid. In addition, decreased the abundance of ruminal methanogens, inhibited CH_4_ synthesis and metabolism.

## 6 Prospect

Competency in nutritional science is essential for veterinarians to be able to promote and maintain good health in all types of livestock. In addition, Veterinarians, technicians, and students must consider using social media platform as an innovative educational tool (Edlira et al., 2023; Muca et al., 2023). Which is importance of effective teaching methods in shaping knowledgeable students and proficient veterinarians. As the social media landscape evolves. Which can result in a significant platform for knowledge sharing, collaboration, and engagement among veterinary professionals and students. Finally, CK is a kind of high nutrient roughage resource with local characteristics, has little food-feed competition, and could be one of the promising tools for small and marginal farmers to reduce the negative environmental impact of livestock production while improving the productivity.

## Data availability statement

The original contributions presented in the study are included in the article/supplementary material, further inquiries can be directed to the corresponding authors.

## Author contributions

XN: Methodology, Software, Writing – original draft. YX: Methodology, Software, Writing – original draft. JW: Data curation, Writing – review & editing. LB: Data curation, Writing – review & editing. YX: Data curation, Writing – review & editing. SZ: Methodology, Writing – review & editing. MS: Methodology, Writing – review & editing. JY: Methodology, Writing – review & editing. DL: Funding acquisition, Investigation, Project administration, Resources, Supervision, Validation, Visualization, Writing – review & editing. YL: Formal analysis, Investigation, Supervision, Validation, Visualization, Writing – review & editing.

## Footnotes

^1^ https://cloud.majorbio.com/
