# Corrigendum: Effects of *Caragana korshinskii* tannin on fermentation, methane emission, community of methanogens, and metabolome of rumen in sheep

**DOI:** 10.3389/fmicb.2024.1433047

**Published:** 2024-06-17

**Authors:** Xiaoyu Niu, Yuanyaun Xing, Jingyao Wang, Lili Bai, Yongfang Xie, Shouqian Zhu, Mei Sun, Jing Yang, Dabiao Li, Yuanyuan Liu

**Affiliations:** ^1^Inner Mongolia Key Laboratory of Animal Nutrition and Feed Science, College of Animal Science, Inner Mongolia Agricultural University, Hohhot, China; ^2^College of Science, Inner Mongolia Agricultural University, Hohhot, China

**Keywords:** condensed tannin, sheep, nutrient digestibility, volatile fatty acids, methane emission

In the published article, there was an error in the Funding statement. The Basic Research Fund for Universities in Inner Mongolia Autonomous Region was omitted. The correct Funding statement appears below.

